# Implementation of Cone Beam Computed Tomography-Guided Online Adaptive Radiotherapy for Challenging Trimodal Therapy in Bladder Preservation: A Report of Two Cases

**DOI:** 10.7759/cureus.66993

**Published:** 2024-08-16

**Authors:** Samyak Jain, John S Peterson, Vladimir Semenenko, Gage Redler, G. Daniel Grass

**Affiliations:** 1 College of Medicine, University of South Florida, Tampa, USA; 2 Department of Radiation Oncology, H. Lee Moffitt Cancer Center & Research Institute, Tampa, USA

**Keywords:** trimodality therapy, artificial intelligence, re-irradiation, muscle-invasive bladder cancer, online adaptive radiotherapy, bladder preservation

## Abstract

Muscle invasive bladder cancer (MIBC) is an aggressive disease with a high risk of metastasis. Bladder preservation with trimodality therapy (TMT) is an option for well-selected patients or poor cystectomy candidates. Cone beam computed tomography (CBCT)-guided online adaptive radiotherapy (oART) shows promise in improving the dose to treatment targets while better sparing organs at risk (OARs). The following series presents two cases in which the capabilities of a CBCT-guided oART platform were leveraged to meet clinical challenges. The first case describes a patient with synchronous MIBC and high-risk prostate cancer with challenging target-OAR interfaces. The second recounts the case of a patient with a history of low dose rate (LDR) brachytherapy to the prostate who was later diagnosed with MIBC and successfully treated with CBCT-guided oART with reduced high-dose volume bladder targeting. To date, both patients report minimal side effects and are without disease recurrence. These cases illustrate how CBCT-guided online adaptive systems may efficiently aid radiation oncologists in treating patients with more complex clinical scenarios who desire bladder-sparing therapy.

## Introduction

Bladder cancer is a particularly aggressive disease, and of the estimated 80,000 new cases diagnosed annually in the United States, about one in four will be muscle-invasive bladder cancer (MIBC), which carries a 50% risk of metastasis [1]. Recent studies have demonstrated that, in properly selected patients, the clinical outcomes for MIBC treated with trimodal therapy (TMT) are similar to a series of neoadjuvant chemotherapy followed by cystectomy [2]. TMT, which is comprised of intact bladder tumor resection followed by concurrent radiotherapy and chemotherapy, allows an opportunity for bladder preservation. This is of particular importance as many patients opt to preserve their bladder given the morbidity and quality of life detriments associated with cystectomy [3].

Effective delivery of radiotherapy relies on precise imaging and dose calculation within the body. The positional and volumetric variability of the bladder as it fills combined with its proximity to surrounding radiosensitive organs at risk (OARs), such as the small bowel, make it a particularly challenging treatment target. This is further complicated when patients have received prior radiation within the target region, which in many instances is a contraindication to bladder preservation.

Online adaptive radiotherapy (oART) addresses many of these issues by providing an opportunity to plan each treatment fraction based on the anatomy of the day (i.e., accounting for variability in daily bladder fill) while avoiding delays if there are extremes in target or OAR variation [4-6]. Technological advances, including the Ethos™ (Varian Medical Systems, Palo Alto, CA) cone beam computed tomography (CBCT)-guided linear accelerator, show promise for delivering oART to maintain target coverage and spare OARs [7,8]. Independent evaluation of artificial intelligence (AI)-mediated contours automatically generated by Ethos™ has already been initiated for specific disease sites, including bladder cancer [8,9]. These results show acceptable, and in some cases, improved, accuracy that did not require laborious hands-on editing. However, continued improvements in autocontouring algorithms and image quality with HyperSight advanced CBCT warrant additional investigations to continue to evaluate the capabilities and limitations of these tools and how they impact the ability of Ethos™ to adapt treatment to the anatomy of the day [10-12].

Given the inherent challenges of bladder irradiation, here, we present two cases of patients treated on the Ethos™ system using CBCT-guided oART with bladder-sparing intent.

## Case presentation

Case 1

**Figure 1:**
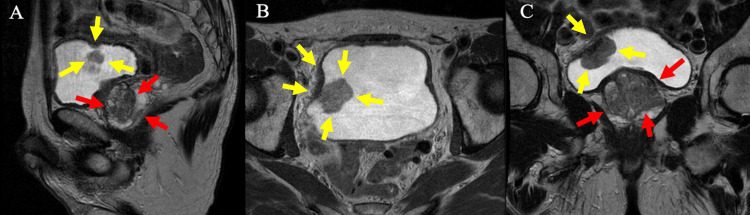
Multiparametric prostate magnetic resonance imaging (mpMRI) of the prostate showing prostate and bladder masses A. sagittal, B. axial, C. coronal; images of a mpMRI of the prostate (T2-weighted sequence). Yellow arrows indicate bladder tumor and red arrows indicate suspected prostate cancer.

A 68-year-old male, never-smoker, with a history of hypercholesterolemia and diverticulosis presented with synchronous prostate and bladder cancer. His prostate-specific antigen (PSA) had been followed for several years and gradually rose to 12.0 ng/mL. He underwent a multiparametric prostate magnetic resonance imaging (MRI) scan (mpMRI) that demonstrated a 58 cc gland with a 3.4 cm x 1.9 cm x 2.3 cm T2-hypointense prostate imaging-reporting and data system (PI-RADS) 5 lesion in the left peripheral zone with associated restricted diffusion and enhancement that crossed the midline and abutted the capsule with concern for extracapsular extension (Figure 1). No seminal vesicle invasion or pelvic adenopathy was appreciable. Incidentally, a 2.8 x 3.4 cm pedunculated mass was seen along the right superolateral wall of the bladder without extravesical extension. Computed tomography (CT) urogram confirmed the intraluminal mass along the right bladder wall without evidence of upper tract or metastatic disease.

He then underwent a cystoscopy, transurethral resection of bladder tumor (TURBT), and a 12-core transperineal prostate biopsy. The cystoscopy identified a prominent median prostate lobe and the right bladder wall mass without any satellite tumors. There was no evidence of diffuse erythema or tumor involvement of the ureteral orifices. The bladder mass was resected and the pathology from the TURBT specimen confirmed high-grade papillary urothelial carcinoma with invasion into the muscularis propria. The prostate biopsies demonstrated Gleason score of 4+4=8 in three cores and 4+3=7 in four cores; intraductal features were noted. Additional work-up with an ^18^fluorine-DCFPyL prostate-specific membrane antigen (PSMA) scan demonstrated no abnormal uptake within the prostate or metastatic disease, regionally or distantly. Therefore, his prostate and bladder cancer staging were T1c (T3a on MRI) N0 M0 and cT2 N0 M0, respectively.

He was seen in multidisciplinary consultation with a urologic oncologist, radiation oncologist, and medical oncologist and presented the treatment options of neoadjuvant chemotherapy followed by cystoprostatectomy with ileal diversion or androgen deprivation therapy (ADT) and TMT for organ preservation. He declined extirpative surgery as his primary management. Given his high-risk prostate cancer, he was started on ADT with degarelix and then underwent repeat TURBT, which noted residual high-grade urothelial carcinoma. The medical oncologist recommended weekly cisplatin during radiation for the MIBC.

He then came in for a CT simulation in a supine position with arms on his chest, and a customized mold was used for immobilization during radiotherapy. He was instructed to have an empty rectum and to void his bladder prior to his non-contrast scan. The scan volume was from the diaphragm through the mid-femurs in 3-mm slices; IV and oral contrast were used to highlight the anatomy. An additional scan was taken 10 minutes after the contrast scans to assess for patient-specific bladder filling variability. 

Given the synchronous high-risk prostate cancer and MIBC, the clinical target volumes (CTVs) included the prostate plus seminal vesicles (target delineation aided by the registered T2-weighted mpMRI of the pelvis); the whole empty bladder plus any evidence of extravesical extension; and the bilateral obturator, internal iliac, external iliac and presacral lymph nodes. The superior border of the CTV nodal field was taken to the common iliac vessel bifurcation given the high-risk prostate cancer. Customized planning target volume (PTV) expansions were used for the prostate and bladder separately and are described in Table 1. The OARs included the rectum, separately defined sigmoid, and bowel bag within 3 cm of the PTV. The decision was made to employ a hypofractionated approach given the BC2001 trial and the various moderate-hypofractionated radiotherapy trials in prostate cancer that have demonstrated safety and efficacy [13,14]. The prescription goals were as follows: 5500 cGy to the bladder, 5500-6000 cGy to the prostate and seminal vesicles, and 4200 cGy to the nodal fields using simultaneous integrated boost (SIB) methods over the course of 20 fractions with concurrent weekly cycles of cisplatin 40 mg/m^2^. He was treated with intensity-modulated radiotherapy (IMRT) using CBCT-guided oART on Ethos™.

**Table 1:** Derived structures for Case 1 To facilitate optimal dosimetry and ensure an efficient online adaptive process with minimal manual intervention in Case 1, the following derived structures were used within the Ethos™ planning directive. ("S," “I," “A," “P," “R," and “L” refer to the superior, inferior, anterior, posterior, right, and left directions, respectively.)

Structure name	Derivation	Purpose
CTV_Bladder	Bladder	Influencer uses OAR to derive targets directly
PTV_Nodes	(CTV_Nodes + 3mm) × (Body – 3mm)	MD-defined expansions
PTV_Prostate	[CTV_Prostate + 7mm(L/R/A) + 5mm(S/I) + 4mm(P)] × (Body – 3mm)	MD-defined expansions
PTV_Bladder	[CTV_Bladder + 12mm(A) + 10mm(S) + 8mm(L/R/I))]× (Body – 3mm)	MD-defined expansions (incorporating potential bladder fill during adaptive process)
opti_PTV_Bladder1	PTV_Bladder – PTV_Prostate	Optimize to avoid excessive hot spots in bladder while pushing more dose to prostate
opti_PTV_Bladder2	PTV_Bladder – Bowel	Selectively optimize target not overlapping with bowel
opti_PTV_Nodes1	PTV_Nodes – (PTV_Bladder + 25mm) – (PTV_Prostate + 25mm)	Optimize for homogeneity on low dose target with fall off from high dose targets
opti_PTV_Nodes2	PTV_Nodes – Bladder_Prostate_PTVs_Exp	Similar to “opti_PTV_Nodes1” but specific to low dose target portions superior to high dose targets
Bladder_Prostate_PTVs_Exp	[Bladder_Prostate_PTVs + 30mm(L/R/A/P/I) + 5mm(S)] × Body	Used to create “opti_PTV_Nodes2”
Bladder_Prostate_PTVs	PTV_Bladder + PTV_Prostate	Used to create “Bladder_Prostate_PTVs_Exp”
opti_PTV_Prostate_Rim	PTV_Prostate – (CTV_Prostate + 2mm)	Optimize to avoid hot spots in ‘PTV_Prostate” outside of “CTV_Prostate”
opti_Bowel_in_PTVBladder	Bowel × PTV_Bladder	Optimize to put more weight avoiding excessive dose to bowel overlapping with target
Bowel	temp_AI_Bowel – Sigmoid	True bowel OAR
temp_AI_Bowel	NA	Used to allow Ethos™ AI to contour all bowel (by definition, including all non-rectum bowel) and then derive true bowel for optimization as this minus manually contoured sigmoid

Treatment Planning

Table 1 details the structural derivations used within Ethos™. This was done to minimize manual edits at the machine during online adaptation and to generate appropriate planning structures that optimize dose distributions. For example, variations on targets are created with “opti” designation to separate out targets receiving different prescribed dose levels. These help to ensure optimal conformality of each prescribed dose level to the respective associated targets. Table 1 also contains details on how the AI autocontouring tools for the pelvis within Ethos™ could be used to contour the machine-defined bowel (referred to as temp_AI_Bowel), which included sigmoid colon and all small bowel, while still efficiently separating out the sigmoid colon each day to allow separate dose goals that accounted for variable radiosensitivity between these OARs.

Table 2 shows how the Ethos™ planning directive was developed to generate the treatment plan, including the structures given dosimetric goals for optimization, the physician-defined dosimetric goals for both targets and OARs, the associated clinical goals within the Ethos™ plan, and respective priorities. The values achieved for the reference plan when using this planning approach are also shown in parentheses. Note that, in some cases, the physician-defined goals were simply unobtainable in the initial reference plan due to patient anatomy and prioritized target coverage. Overall, the top priority was to respect the "Dmax" (maximum dose to a 0.03cc subvolume) for the bowel, sigmoid, and rectum. The next priority was to homogeneously cover the CTV_Bladder (clinical target volume of the bladder) with 5500 cGy. The prostate volume receiving at least 5500 cGy was maximized while also pushing as much as 6000 cGy dose into this target to achieve an enhanced therapeutic dose. The nodal targets were also treated as homogeneously as possible to 4200 cGy.

**Table 2:** Dosimetric goals for Case 1 For Case 1, the structures used for Ethos™ plan optimization to best achieve physician-defined goals and how these were assigned prioritized clinical goals in the Ethos™ planning directive. Structures without physician clinical goals are helper structures generated solely for treatment planning. Note: All maximum and minimum doses are to 0.03 cc volumes. The prioritization of each dosimetric goal is enclosed in curly brackets ({}). *Failed goal in the reference plan that was deemed to be acceptable in order to achieve conflicting goals.

Structure name	Physician goal(s) (achieved reference dosimetry)	Ethos™ goal(s)
PTV_Bladder	D95% ≥ 5225 cGy (5329), Dmax ≤ 6300 cGy (6125)	{2} D95% ≥ 5500 cGy, {4} Dmin ≥ 5225 cGy
CTV_Bladder	D95% ≥ 5225 cGy (5457), Dmax ≤ 5800 cGy (5774)	{1} Dmax ≤ 5600 cGy, {3} V5500 cGy ≥ 99%, {3} V5600 cGy ≤ 40%
PTV_Prostate	D95% ≥ 5225 cGy (5262), Dmax ≤ 6600 cGy (6528)	{1} D95% ≥ 5500 cGy, {3} Dmax ≤ 6300 cGy, {4} V5500 cGy ≥ 97%
CTV_Prostate	NA	{3} Dmean ≥ 6000 cGy, {3} V5500 cGy ≥ 99%
PTV_Nodes	D95% ≥ 3990 cGy (4193)	{1} D95% ≥ 4200 cGy, {4} V4000 cGy ≥ 98%, {4} V4620 cGy ≥ 10%
CTV_Nodes	NA	{3} V4200 cGy ≥ 99%
Rectum	Dmax ≤ 5600 cGy (5485), V2500 cGy ≤ 50% (45.9), V4000 cGy ≤ 30% (22.2), V4700 cGy ≤ 10% (11.8)*, V5400 cGy ≤ 5% (0.5), V5500 cGy ≤ 1% (0.1)	{1} Dmax ≤ 5600 cGy, {1} V4700 cGy ≤ 10%, {2} V2500 cGy ≤ 50%, {4} Dmean ≤ 2500 cGy, {4} V5500 cGy ≤ 1%, {4} V4000 cGy ≤ 30%
Bowel	Dmax ≤ 5800 cGy (5765), V5700 cGy ≤ 2cc (1.0), V5500 cGy ≤ 3cc (20.3)*, V5200 cGy ≤ 20cc (58.6)*, V4300 cGy ≤ 40cc (125.1)*, V4000 cGy ≤ 150cc (161.9)*, V2500 cGy ≤ 300cc (370.6)*	{1} Dmax ≤ 5800 cGy, {2} D3.00cc ≤ 5500 cGy, {2} V2500 cGy ≤ 300 cc, {4} V5500 cGy ≤ 3.00cc, {4} Dmean ≤ 2000 cGy
Sigmoid	Dmax ≤ 5800 cGy (5717), V5700 cGy ≤ 3cc (0.0), V5500 cGy ≤ 10cc (4.0), V5000 cGy ≤ 20cc (11.7), V3000 cGy ≤ 45% (42.9)	{1} Dmax ≤ 5800 cGy, {2} V3000 cGy ≤ 40%, {3} D10.0cc ≤ 5500 cGy, {4} Dmean ≤ 3200 cGy
Femoral Heads	Dmax ≤ 4200 cGy (3383), V4000 cGy ≤ 15% (0.0), V3000 cGy ≤ 20% (0.1)	{4} Dmax ≤ 4200 cGy
opti_PTV_Bladder2	NA	{1} D95% ≥ 5500 cGy
opti_Bowel_in_PTVBladder	NA	{1} Dmax ≤ 5500 cGy, {2} V5300 cGy ≥ 95%
opti_PTV_Prostate_Rim	NA	{3} Dmax ≤ 6300 cGy
opti_PTV_Nodes1	NA	{3} V4410 cGy ≤ 5%, {4} Dmax ≤ 4620 cGy
opti_PTV_Nodes2	NA	{3} V4410 cGy ≤ 1%, {4} Dmax ≤ 4410 cGy
opti_PTV_Bladder1	NA	{3} Dmax ≤ 6050 cGy

**Figure 2:**
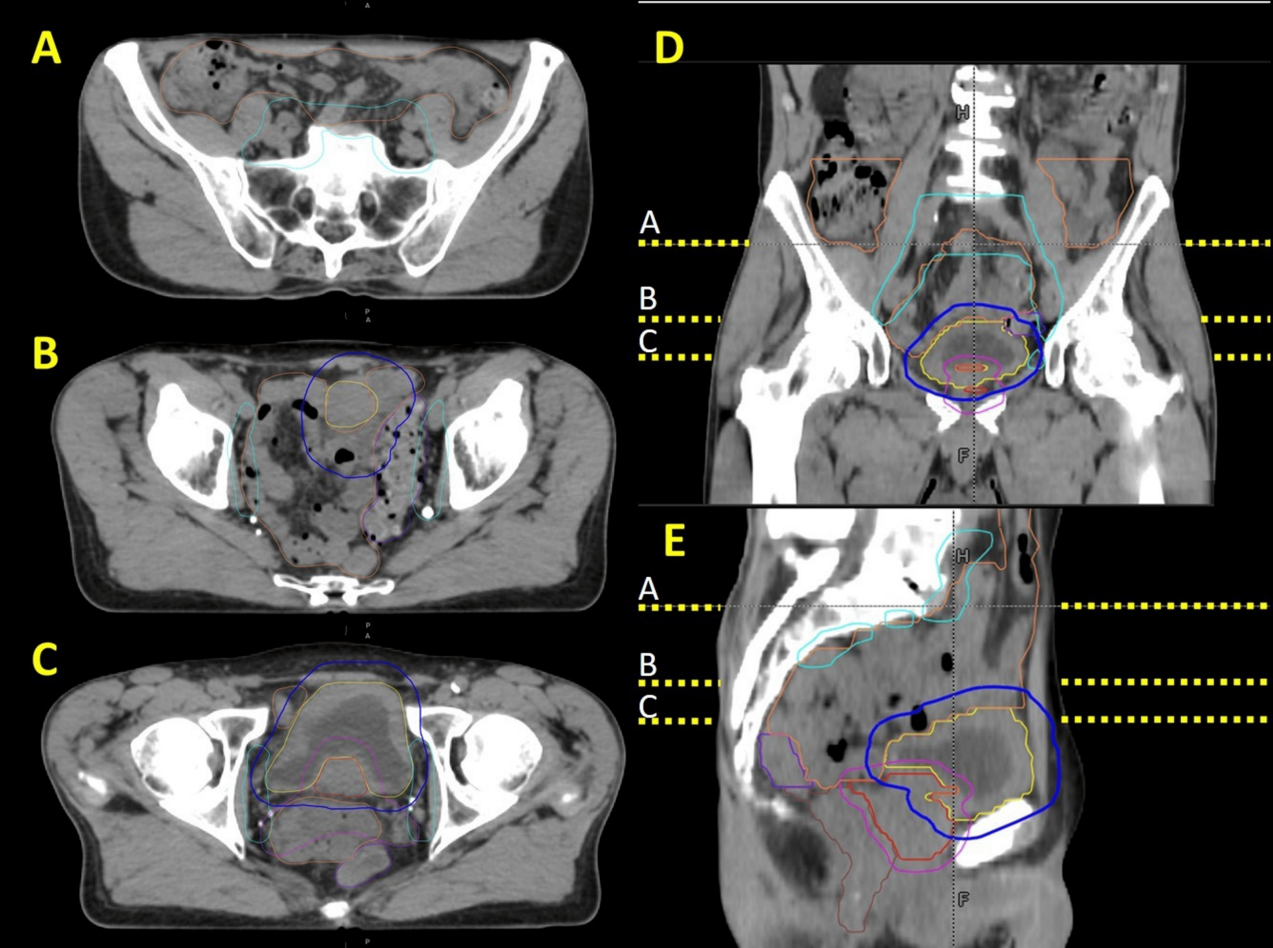
Depiction of patient anatomy on initial planning simulation CT scan in Case 1 Certain structures are shown, including CTV_Prostate (red),  CTV_Bladder (yellow), PTV_Prostate (magenta), PTV_Bladder (blue), PTV_Nodes (cyan), Bowel (orange), Sigmoid (purple), and Rectum (brown). (A)-(C) show axial slices moving from superior to inferior in the patient and corresponding to their respective yellow dashed lines in the (D) coronal and (E) sagittal views.

Figure 2 depicts the initial planning anatomy for this patient and highlights the complexity of this case (craniocaudal location of the displayed axial slices in Figure 2A-2C is given by dashed yellow lines in Figure 2D-2E). Since this patient had synchronous prostate and bladder cancer with a nodal volume, the sheer quantity and volume of treatment targets made it challenging to meet dose constraints and avoid OAR toxicity. The patient's small body habitus and bowel configuration further complicated planning since certain bowel loops fell posterior and in close apposition to the prostate and seminal vesicles, effectively surrounding all targets. This plan was essentially a three-dose-level SIB with levels of 6000, 5500, and 4200 cGy in 20 fractions. The initial plan selected with optimal dosimetry and clinical priorities consisted of 17 equally spaced, fixed-field IMRT beams all with collimator angles of 10^o^. The total monitor units (MUs) were 3315.2 distributed between these beams.

Treatment Adaptation and Delivery

For the online adaptive treatments, the patient was set up with minimal immobilization in a supine position using laser localization to approximately align to marks placed during CT simulation. The patient was then imaged with the Ethos™ six-second Hypersight CBCT incorporating iterative reconstruction with Acuros scatter correction. The influencers were then generated via AI contouring. These included the rectum, bladder (used to generate CTV_Bladder and derive PTV_Bladder), and the temp_AI_Bowel contour (defined as all bowel, including sigmoid). Once the influencers were deemed accurate, the targets and all other contours were generated via influencer-guided deformable registration.

Next, the sigmoid was manually edited, cropping it out of temp_AI_Bowel to derive the true bowel. The CTV_Nodes was visually assessed, and if it did not match the daily patient anatomy, it was rigidly propagated and aligned; this is essentially equivalent to standard 3D image matching to the bone with image-guided radiotherapy (IGRT). The CTV_Prostate was manually edited; in some instances, this could be rigidly propagated and aligned if the borders were unclear on the daily CBCT. Once the contours were all approved, the remaining structures were derived and plans, both scheduled and adapted, were generated for evaluation.

The physician and physicist would review and select the optimal plan. Once this plan was approved by both parties for treatment, a verification CBCT was acquired to ensure that no gross patient repositioning occurred and that bowel gas and bladder fill during the contour and plan adaptation did not cause targets to change appreciably. MobiusAdapt was used as the independent calculation for IMRT plan quality assurance (QA). Finally, the treatment selected for that day was delivered to the patient. Overall, the time from initial CBCT acquisition to treatment completion across the 20 treatment fractions for this patient averaged 36.1 minutes (range: 24.2, 51.0).

For this case, online plan adaptation with Ethos™ was found to be extremely beneficial dosimetrically. The adapted plan was selected over the scheduled plan in 100% of the treatment sessions. Table 3 shows the comparison between a subset of critical dosimetric goals for the initial reference plan and average values for scheduled versus adapted plans and the mean percent differences between scheduled and adapted plans (percent difference = [adapted value - scheduled value]/scheduled value). Without adaptation, the OAR goals were often violated. For example, the Bowel Dmax goal would have been violated in 19 of 20 (95%) sessions with the scheduled plan compared to only one of 20 (5%) with the adapted plan; in that single instance, the adapted plan still decreased this value by 7.5%. The rectum Dmax would have been violated in all 20 (100%) sessions with the scheduled plan compared to only 5/20 (25%) sessions with the adapted plan; for those five, the value still decreased each time with adaptation by an average of 8.4%. The Sigmoid Dmax would have been violated in seven of 20 (35%) sessions with the scheduled plan versus four of 20 (20%) with the adapted plan; only two of the four were still decreased with adaptation. Adapting also simultaneously improved target coverage. For example, the PTV_Prostate D95% goal would have failed in four out of 20 (20%) sessions with the scheduled plan versus 0 out of 20 (0%) with the adapted plan. The PTV_Bladder D95% would have failed in 16 out of 20 (80%) scheduled plans versus none (0%) with the adapted plan. The PTV_Nodes D95% goal would have failed in 18 out of 20 (90%) scheduled plans versus three out of 20 (15%) with the adapted plan; for all three of these, plan adaptation still improved this metric, with an average gain of 1.9%. 

**Table 3:** Comparison of dosimetric outcomes in Case 1 For Case 1, the summary of critical dosimetric objectives comparing physician goals with achieved values in the initial reference plan, scheduled plan, and adapted plan, as well as the average percent difference for those objectives achieved in the scheduled vs. adapted plans. All maximum doses are to 0.03 cc volumes.

Dosimetric objective	Goal per fraction (cGy)	Dose per fraction in the reference plan (cGy)		Mean dose per fraction in the scheduled plan (Range)	Mean dose per fraction in the adapted plan (Range)	Mean percent difference (Range)
Bowel Dmax	≤ 290.0	288.3	316.6 (293.6, 339.6)		288.1 (285.7, 290.5)	-8.4 (-17.7, 0.9)
Rectum Dmax	≤ 280.0	274.3	311.4 (302.9, 319.9)		278.3 (275.6, 281.0)	-10.6 (-13.3, -7.9)
Sigmoid Dmax	≤ 290.0	285.9	290.7 (277.3, 304.1 )		284.3 (271.1, 297.5)	-1.9 (-9.1, 5.3)
PTV_Prostate D95%	≥ 261.3	263.0	266.1 (261.3, 270.9)		272.0 (269.5, 274.5)	2.2 (0.5, 3.9)
PTV_Bladder D95%	≥ 261.3	266.0	248.8 (213.1, 284.5)		266.8 (264.0, 269.6)	4.1 (0.9, 7.3)
PTV_Nodes D95%	≥ 199.5	210.0	187.5 (176.3, 198.7)		208.0 (201.5, 214.5)	11.4 (2.9, 19.9)

This patient is currently three months status post-treatment and has no evidence of recurrent disease on CT imaging, cystoscopy, or urine cytology. He reports no changes in urinary or bowel quality of life to date. PSA is undetectable.

Case 2

An 87-year-old male, former smoker with multiple comorbidities, including hypertension, hyperlipidemia, atrial fibrillation, and deep vein thrombosis complicated by a pulmonary embolism (on rivaroxaban) was diagnosed with MIBC. He ultimately was deemed a poor cystectomy candidate. He also had a history of low-risk prostate cancer, which was treated definitively with low-dose-rate (LDR) brachytherapy using ^125^Iodine 20 years prior.

He initially presented with hematuria and clot formation resulting in urinary obstruction. CT urogram demonstrated a 2.8 cm right posterior bladder mass alongside vascular calcifications with no evidence of hydronephrosis, upper tract, or metastatic disease. A review of the LDR seed distribution demonstrated insertion into a prominent median lobe and with proximity to the bladder neck and urethra interface. CT chest was negative for metastatic disease. Cystoscopy demonstrated a solitary 3-cm tumor along the right lateral wall with no involvement of the ureteral orifice. Pathology from the TURBT demonstrated high-grade urothelial carcinoma with invasion into the muscularis propria.

Upon clinical evaluation, he reported an American Urological Association urinary symptom score (AUA) of 13 with a repeat cystoscopy demonstrating the prior resection scar was along the right lateral wall away from the trigone and bladder neck. The multidisciplinary discussion presented him with options of organ preservation with TMT or repeat TURBTs with or without intravesical therapy as needed. He elected to move forward with TMT.

The simulation process was performed in the same manner as the patient in Case 1, except with the omission of IV and oral contrast. Given his prior LDR prostate brachytherapy history and distribution of seeds, the treatment approach was to consider covering the right bladder wall and resection bed with 5500 cGy with a reduced dose to the uninvolved bladder and trigone and bladder neck in 20 fractions with CT-guided oART on the Ethos™. This reduced high-dose volume approach was evaluated as a treatment arm in the BC2001 trial without detriment in patient outcomes [13]. Coverage of the lymph nodes was omitted. He also received concurrent biweekly gemcitabine (27 mg/m^2^).

Treatment Planning

Table 4 details the structure derivations used within Ethos™ for this case to minimize manual edits and generate appropriate planning structures and optimal dose distributions. The anisotropic CTV expansions were determined by the attending physician based on the tumor bed location and the bladder proper. The opti target structures were cropped a certain distance away from the avoidance region and were empirically determined to create sufficient space for limiting the maximum dose in the avoidance region to 4100 cGy while also delivering 5500 cGy to the target. The anisotropic expansions to PTV were determined by the physician based on surgical bed location and presumed bladder fill.

**Table 4:** Derived structures for Case 2 To facilitate optimal dosimetry and ensure an efficient online adaptive process with minimal manual intervention in Case 2, the following derived structures were used within the Ethos™ planning directive. ("S," “I," “A," “P," “R," and “L” refer to the superior, inferior, anterior, posterior, right, and left directions, respectively.)

Structure Name	Derivation	Purpose
opti_Avoid	Prostate + 10mm(S) + 5mm(A)	Conservative approximation for urethra/trigone avoidance region
opti_Avoid_Exp	opti_Avoid + 10mm(L/R/A/P) + 5mm(S/I)	Expansion of avoidance to crop from targets to allow reasonable dose fall off
Brush_RtBladderWall	NA	Manual structure for drawing about 10 mm thick rind on right bladder wall extending slightly outside of the bladder
tempCTV	Brush_RtBladderWall ∩ Bladder	Create right wall of bladder (removing extension outside of bladder)
CTV_RtBladderWall	tempCTV – opti_Avoid	Create right wall of bladder without urethra/trigone overlap
opti_CTV_RtBladderWall	CTV_RtBladderWall – opti_Avoid_Exp	Create region of CTV that can reasonably be covered with prescription dose while sparing urethra/trigone
tempPTV	CTV_RtBladderWall + 5mm(S/I/P) + 8mm(L) + 10mm(R/A)	Initial expansion to create PTV allowing for bladder fill uncertainty during online adaptation
PTV_RtBladderWall	tempPTV – opti_Avoid	Create right bladder wall with PTV margin but without urethra/trigone overlap
opti_PTV_RtBladderWall	PTV_RtBladderWall - opti_Avoid_Exp	Create a region of PTV that can be reasonably covered with a prescription dose while sparing urethra/trigone

**Figure 3:**
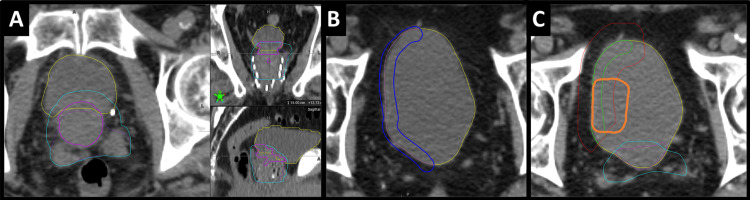
Depiction of critical structure derivations in Case 2 The above image shows how the volumes of the final targets and avoidance regions were generated. (A) Relatively inferior axial slice with the bladder (yellow), true urethra/trigone (magenta), and the resultant opti_Avoid (cyan) generated on the initial planning CT. Coronal and sagittal views are displayed as well, with the high-intensity/high-Z prostate seeds visible from previous LDR brachytherapy. (B) Axial slice showing the bladder (yellow), Brush_RtBladderWall (blue) that was manually drawn to about 10 mm thick on the right side of the bladder, and the resulting tempCTV for the right bladder wall to be treated. (C) shows the final targets: opti_CTV_RtBladderWall (green) and opti_PTV_RtBladderWall (red) that have been derived from tempCTV and cropped away from the opti_Avoid (cyan). The approximate original tumor location (delineated via registration of pre-op MRI) is shown (orange) to demonstrate the previously involved bladder wall region is encompassed by the derived targets.

Figure 3 shows a visual representation of these structures on the initial planning CT with a comparison to the approximate pre-operative tumor location.

Table 5 shows how the Ethos™ planning directive was developed to generate the treatment plan. As in Case 1, patient anatomy and prioritized sparing of the previously irradiated area made some physician-defined goals impossible. Overall, the goal was to adaptively cover the resection bed along the right bladder wall with a dose cloud that accounted for bladder fill changes while simultaneously avoiding the urethra/trigone region due to the previous dose delivered to the prostate via LDR brachytherapy. This plan was developed to efficiently adapt this avoidance region for bladder and rectal changes, which could subsequently alter prostate and urethra trigone positioning. The initial plan selected with optimal dosimetry and clinical priorities consisted of nine equally spaced, fixed-field IMRT beams all with collimator angles of 10^o^; total MUs were 1976.4.

**Table 5:** Dosimetric goals for Case 2 For Case 2, the structures used for Ethos™ plan optimization to best achieve physician-defined goals and how these were assigned prioritized clinical goals in the Ethos™ planning directive. Structures without physician clinical goals are helper structures generated solely for treatment planning. Note: All max and min doses are to 0.03 cc volumes. The prioritization of each dosimetric goal is enclosed in curly brackets ({}). *Failed goal in the reference plan that was deemed to be acceptable in order to achieve conflicting goals.

Structure mame	Physician goal(s) (achieved reference dosimetry)	Ethos™ goal(s)
PTV_RtBladderWall	V5500cGy ≥ 95% (85.6)*, Dmax ≤ 5885 cGy (5830)	{3} Dmin ≥ 4100 cGy
CTV_RtBladderWall	V5500cGy ≥ 99% (88.5)*	{3} Dmin ≥ 4100 cGy
Bladder	Dmax ≤ 5800 cGy (5717), V5600 cGy ≤ 40% (23.6)	{4} V5600 cGy ≤ 40.0%
Urethra_Trigone	Dmax ≤ 5000 cGy (4030), V3000 cGy ≤ 60% (10.0), V2200 cGy ≤ 20cc (2.6)	
opti_Avoid	Dmax ≤ 4100 cGy (4070), V2000 cGy ≤ 20cc (16.0)	{1} Dmax ≤ 4100 cGy, {2} V2000 cGy ≤ 20.0cc
Rectum	Dmax ≤ 5600 cGy (2236), V2500 cGy ≤ 50% (0.0), V4000 cGy ≤ 30% (0.0), V4700 cGy ≤ 10% (0.0), V5400 cGy ≤ 5% (0.0), V5500 cGy ≤ 1% (0.0)	
Bowel	Dmax ≤ 5800 cGy (5578), V5700 cGy ≤ 2cc (0.0), V5500 cGy ≤ 3cc (0.5), V5200 cGy ≤ 20cc (2.1), V4300 cGy ≤ 40cc (6.5), V4000 cGy ≤ 150cc (8.4), V2500 cGy ≤ 300cc (25.0)	{1} Dmax ≤ 5800 cGy, {2} V5700 cGy ≤ 2.0cc, {3} V5200 cGy ≤ 20.0cc
Femoral Heads	Dmax ≤ 4200 cGy (2369), V4000 cGy ≤ 15% (0.0), V3000 cGy ≤ 20% (0.0)	{4} Dmax ≤ 4200 cGy
opti_PTV_RtBladderWall	NA	{1} V5500 cGy ≥ 95.0%, {2} Dmax ≤ 5775 cGy, {2} D95% ≥ 5225 cGy
opti_CTV_RtBladderWall	NA	{1} V5500 cGy ≥ 99.9%

Treatment Adaptation and Delivery

Similar to Case 1, the patient was imaged with the Ethos™ six-second Hypersight CBCT incorporating iterative reconstruction with Acuros scatter correction. Bowel, bladder, and rectal influencers were generated by AI autocontouring and manually edited where needed. Targets and remaining contours were generated next via influencer-guided deformable image registration from the CBCT to the planning CT. Contours were then edited to account for changes in patient anatomy including (1) remaining edits to bowel, bladder, and rectum; (2) prostate edits to generate the avoidance region approximating the urethra/trigone and region of previous RT; and (3) a 10-mm brush was used to manually paint out the right bladder wall, which was then used to auto-generate targets based on pre-defined margins as described above. Once the contours were approved, the scheduled and adapted plans were generated and evaluated, and a verification CBCT was acquired to ensure no major changes in bladder fill. MobiusAdapt was used as the independent calculation for IMRT plan QA. Finally, the treatment selected for that day was delivered to the patient. Overall, the time from initial CBCT acquisition to treatment completion across the 20 treatment fractions for this patient averaged 15.9 minutes (range: 11.6, 23.8).

Here too, online plan adaptation with Ethos™ was found to be very beneficial dosimetrically. The adapted plan was selected over the scheduled plan in all treatment sessions. Table 6 shows a comparison between a subset of critical dosimetric goals for the initial reference plan and average values for scheduled versus adapted plans and the average percent differences between scheduled and adapted plans. Adaptation was critical for achieving adequate target coverage. The opti_CTV_RtBladderWall target would have been below the physician-defined D99% by an average of 3.1%, and up to 13.5%, in all 20 fractions with the scheduled plan. By contrast, only three of 20 (15%) failed with plan adaptation, each by only 1 cGy or 0.4% lower than the D99% physician goal. Likewise, the opti_PTV_RtBladderWall was on average 5.1% below the physician-defined D95% in all 20 fractions with the scheduled plan, while only 6 of 20 (30%) fractions did not meet this goal with the adaptive plan, each by only 1 cGy or 0.4%. At the same time, OARs and the avoidance region were better spared with plan adaptation. The bowel Dmax objective was never violated for either the scheduled or adapted plan, demonstrating overall favorable anatomical geometry. It was, however, decreased in 18 of 20 (90%) fractions with adaptation (one was equal and one increased by 2.9%). The opti_Avoid structure Dmax would have failed to meet the objective in 19 of 20 (95%) fractions with the scheduled plan versus in 0 of 20 fractions (0%) with the adapted plan. Note, the V5500 cGy metrics were not included in the table or assessed for these percent differences due to near zero values in the scheduled plan, which would result in dividing by very small numbers. However, on average, the opti_CTV_RtBladderWall and opti_PTV_RtBladderWall coverage goals (V5500cGy ≥ 99% and V5500cGy ≥ 99%, respectively) were far superior for the adapted plan versus the scheduled plan: 99.2 (range: 98.8, 99.6)% versus 23.3 (range: -6.2, 52.8)% and 94.6 (range: 94.0, 95.2)% versus 23.9 (range: 2.4, 45.4)% for the opti_CTV_RtBladderWall and opti_PTV_RtBladderWall goals, respectively.

**Table 6:** Comparison of dosimetric outcomes in Case 2 For Case 2, the summary of critical dosimetric objectives comparing physician goals with achieved values in the initial reference plan, scheduled plan, and adapted plan, as well as the average percent difference for those objectives achieved in the scheduled vs. adapted plans. Note, due to low V5500 cGy values in the scheduled plan, the percent difference was not evaluated for this metric. All maximum doses are to 0.03 cc volumes.

Dosimetric objective	Goal per fraction (cGy)	Dose per fraction in the reference plan (cGy)	Mean dose per fraction in the scheduled plan (Range)	Mean dose per fraction in the adapted plan (Range)	Mean percent difference (Range)
Bowel Dmax	290.0	279.0	281.3 (277.5, 285.1)	259.7 (243.0, 276.4)	-7.6 (-14.2, -1.0)
opti_Avoid Dmax	205.0	203.0	270.6 (246.7, 294.5)	200.1 (198.4, 201.8)	-25.3 (-34.4, -16.2)
opti_CTV_RtBladderWall D99%	275.0	276.0	266.6 (259.4, 273.8)	275.7 (274.7, 276.7)	3.5 (0.5, 6.5)
opti_PTV_RtBladderWall D95%	275.0	275.0	260.9 (253.8, 268.0)	274.7 (274.2, 275.2)	5.4 (2.3, 8.5)

**Figure 4:**
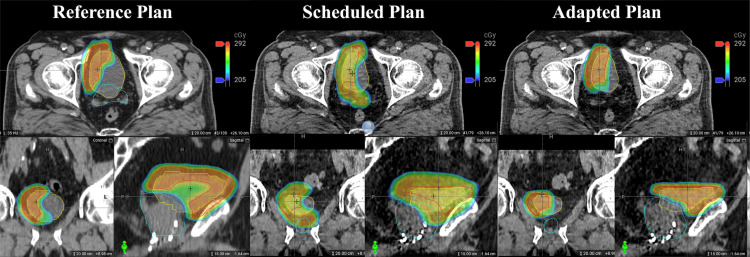
Anatomic comparison of dose distribution in Case 2 For Case 2, sample dosimetry for the reference plan on the planning CT anatomy and scheduled and adapted plans based on the CBCT and anatomy of the day. The lowest portion of the dose colorwash is the Dmax, 0.03 cc goal for the avoidance region (cyan). For each, the top, bottom left, and bottom right sub-images are axial, coronal, and sagittal views, respectively. At treatment, the bladder fill is appreciably less, resulting in normal tissue overdose and target under coverage without plan adaptation.

Figure 4 clearly shows the role of online plan adaptation in this setting as the anatomy of the day has very different bladder fill, thus requiring the high-dose cloud to be adapted accordingly to treat the bladder wall while sparing normal tissue.

This patient is currently three months status post-treatment with no evidence of disease recurrence seen on CT imaging, cystoscopy, and urine cytology. Importantly, he has no complaints of dysuria, hematuria, incontinence, pelvic pain, or bowel changes; his AUA score was relatively unchanged at 15.

## Discussion

Each of the described cases presented unique scenarios that required consideration of advanced radiation delivery. The first case described synchronous prostate and MIBC with unfavorable OAR orientation relative to target structures. Given that hypofractionated regimens of 6000 cGy in 20 fractions are commonly used in prostate cancer and whole empty bladder doses are recommended at 5500 cGy, this created additional hurdles for effective treatment delivery. This was further compounded by the presence of concurrent radiosensitizing chemotherapy and the clinical decision to cover the pelvic nodal fields. Coverage of the entire prostate and pelvic lymph nodes during TMT is at the discretion of the treating physician, with wide variability in protocols, and optimal dose constraints are not currently defined in the setting of hypofractionation. The second case provided a challenge of overlapping radiation doses from prior prostate brachytherapy with a likely dose distribution being substantial at the bladder neck and urethra, especially in a setting of a prominent median lobe. In many instances, prior radiation to the pelvis is a contraindication to bladder radiation, and if suitable, cystectomy is preferred. 

In both cases, the use of the Ethos™ system for CBCT-guided oART allowed for accurate treatment delivery and daily adaptation to anatomic variability. The first case exhibited the improvement of dosimetric objectives, significantly lowering potential damage to OARs and improving target coverage. The bowel, rectum, and sigmoid Dmax values were lower by an average of 8.4%, 10.6%, and 1.9%, respectively, between the scheduled and adapted plan, indicating less radiation being delivered to OARs with oART planning. Furthermore, dosimetric analysis revealed improvements at targets: PTV_Prostate D95%, PTV_Bladder D95%, and PTV_Nodes D95% increased by an average of 2.2%, 4.1%, and 11.4% respectively. Similar changes were seen in the second case (Bowel Dmax improved by 7.6% on average and opti_CTV_RtBladderWall D99% improved by 3.5% on average), although deviations were seen due to specific patient anatomy variability during the treatment period. Whether oART will provide clinically meaningful benefits is a current focus of several enrolling trials ARTIA-Vesica (ClinicalTrials.gov, NCT05295992) and ARTIA-Bladder (ClinicalTrials.gov, NCT02700227), which are summarized by Livschitz et al. [9,15].

The literature regarding bladder treatment with ART is extensive. Heavy emphasis is placed on the utilization of CBCT and plan-of-the-day treatment for bladder contour adjustment and target variability [9]. Murthy et al. utilized plan-of-the-day ART to analyze the outcomes of an MIBC cohort, which found low levels of toxicity to OARs (3.8% having Grade >3 gastrointestinal toxicity) with about 80% sparing their bladder long-term [16]. In a study by Hafeez et al., ART demonstrated dose coverage improvements to the PTV and minimization of OAR dose [17]. Table 7 provides an overview of the use of oART for the purposes of bladder-directed radiotherapy, which overall supports an improvement in target coverage and OAR protection. The paucity of case reports describing oART utilization in bladder cancer is likely influenced by the novelty of this technology. Azzarouali and colleagues analyzed oART in a cohort of 15 patients with MIBC and found that the use of the Ethos™ resulted in significant dose decreases in OARs [8].

**Table 7:** Summary of current studies on the use of online adaptive radiotherapy (oART) for the treatment of bladder cancer. Data displayed includes author and publication year, the number of patients, prescription data (target, dose, and fraction), and findings for the study [7,8,18,19].

Study	No. of patients	Prescription			Findings	
		Target	Dose (Gy)	No. of Fractions		
Azzarouali et al [7], 2023	15	Bladder and pelvic nodes	40	20	CTV coverage V95%	Less non-tumor tissue received 40 and 55 Gy (p < 0.001)
		Tumor bed	55	20	GTV adjustment	3.9 cm^3^ smaller GTV_AI _then GTV_ref_
					Median on-couch Time	22 minutes
Astrom et al [8], 2022	16	Bladder	64	32	Mean Volume reduction of PTV-T	33.9%
		Pelvic ymph nodes	50	32		
		Positive lymph nodes	50	32	Median oART procedure time	13.9 minutes
Fouroudi et al [18], 2011	27	Bladder	64	32	oART V95 at <99%	2.7% compared to 4.8%
					Difference in mean volume of normal tissue receiving >45 Gy	29% less
					CTV volume decrease	27%
Fouroudi et al [19], 2014	50	Bladder	64	32	Percent of patients with at least one grade 3 acute toxicity	23%
					Percent CBCTs partially outside PTV margins	5.5%

The application of Ethos™ and oART is further illustrated by the two cases within this report, extending the potential range and usage of the ART workflow. In both cases, radiotherapy was delivered with a goal of mitigating toxicity to surrounding normal tissue while concomitantly optimizing target coverage.

## Conclusions

These two cases largely illustrate the effectiveness of CBCT-guided oART on the Ethos™ for bladder cancer treatment. With the target sites and surrounding OARs having variable volumes and shapes for both patients, ART accounted for these frequent changes. The first case demonstrated optimized treatment of synchronous cancers of the bladder and prostate with unfavorable anatomy interfaces using CBCT-guided oART. The second case demonstrated the advantage of utilizing oART to minimize toxicity to a frail patient with prior prostate radiation. The ability to employ oART in bladder cancer treatment, especially in the setting of overlapping dose distributions from prior radiotherapy, is evidenced by the optimized target coverage and OAR dose reduction with this workflow.
